# Myosin VI is expressed in developing ovarian follicles in *Drosophila* but is not essential for effective oogenesis

**DOI:** 10.3389/fcell.2025.1535117

**Published:** 2025-06-02

**Authors:** Robert Lenartowski, Jakub Ostrowski, Anna Suwińska, Anna Richert, Przemysław Zakrzewski, Magdalena Izdebska, Wioletta Arendt, Kathryn G. Miller, Marta Lenartowska

**Affiliations:** ^1^ Department of Cellular and Molecular Biology, Faculty of Biological and Veterinary Sciences, Nicolaus Copernicus University in Toruń, Torun, Poland; ^2^ Centre for Modern Interdisciplinary Technologies, Nicolaus Copernicus University in Toruń, Torun, Poland; ^3^ School of Cellular and Molecular Medicine, Faculty of Life Sciences, University of Bristol, Bristol, United Kingdom; ^4^ Department of Histology and Embryology, Faculty of Medicine, Collegium Medicum in Bydgoszcz, Nicolaus Copernicus University in Toruń, Torun, Poland; ^5^ Department of Biology, Washington University in St. Louis, St. Louis, MO, United States

**Keywords:** binary system, border cell migration, cell/molecular biology, *Drosophila* oogenesis, RNAi, myosin VI mutant, transgenic flies

## Abstract

Myosin VI is the only actin-based motor known to move toward the minus end of actin filaments. This protein is involved in many different cellular processes, such as endocytosis, autophagy, secretion, and regulation of actin organization and dynamics. Myosin VI has also been suggested to play an important role in collective migration of border cells and egg chamber development during *Drosophila* oogenesis. Here we show for the first time that myosin VI is expressed in *Drosophila* germarium as well as in early ovarian follicles, especially in the developing oocyte. As oogenesis progresses, the level of myosin VI in maturing egg chambers decreases, but this protein is present both in the nascent border cell cluster, during its delamination from the epithelium, and then during the early stages of border cell migration. However, we demonstrate that myosin VI deficiency in border cells, or even complete lack of this protein in myosin VI mutant do not inhibit border cell migration. Moreover, deficiency/lack of myosin VI does not cause any serious defects in ovarian morphology, egg chamber morphogenesis, oogenesis, and egg development. Thus we conclude that myosin VI is not a key player in *Drosophila* oogenesis.

## Introduction

**FIGURE 1:**
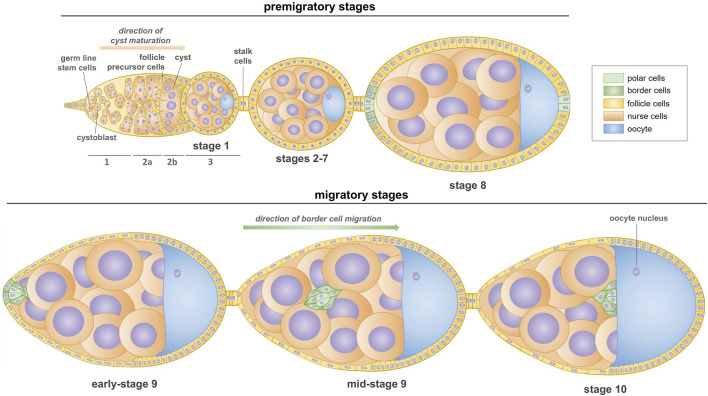
Scheme of oogenesis in *Drosophila* (stages 1–10). The formation of first egg chamber takes place in the germarium, which is made up of three regions. In region 1, germline cells divide asymmetrically and produce cystoblasts which undergo a series of four mitoses with incomplete cytokinesis to form a cyst. Then, 16-cell cysts move through the region 2a and passes the follicle precursor cells which results in encapsulation of cyst by the follicle cells in the region 2b. Stage 1 egg chambers buds off the germarium in the region 3; they consist one oocyte and 15 supporting nurse cells. Subsequent budding egg chambers are connected by the stalk cells. During the next stages (2–7), oocyte increases in size and egg chambers become more oval shaped. At stage 8, two non-motile polar cells at the anterior pole of the egg chamber induce the surrounding epithelial cells (called border cells) to a migratory fate by the specific signaling pathways. At stage 9, the border cell cluster (two non-motile polar cells and few motile border cells, called as the border cells) undergoes a partial epithelial-to-mesenchymal transition and detaches from the epithelium, and then the cluster moves between nurse cells towards the anterior border of the oocyte. Collective migration of border cells ends at the stage 10 of oogenesis, when they reach the oocyte.

Oogenesis in *Drosophila* occurs in ovaries consisting of multiple ovarioles, each forming a chain of developing egg chambers (Bastock and St Johnston, 2008). At the anterior end of the ovariole lies the germarium, a structure housing germline stem cells that initiate the oocyte formation and follicle stem cells that generate the epithelium of the egg chamber (Kirilly and Xie, 2007; Waghmare and Page-McCaw, 2018). Germline stem cells undergo asymmetric divisions forming cysts whose morphology and successive developmental stages differentiates the germarium into three main regions 1, 2a/2b and 3 (Figure 1). Oogenesis begins in region 1, when a germline stem cells divide to produce cystoblasts, which divide four more times to produce 16-cell germline cysts that are connected by ring canals (Huynh and St Johnston, 2004; Jagut et al., 2013). The oocyte differentiates from one of the two cells with four ring canals, which are therefore called the pro-oocytes. Once the 16-cells cyst has formed, it enters the region 2a of the germarium. At this stage, all the cells of one cyst appear similar, but by the time it reaches region 2b, one cell differentiates as an oocyte. It is becoming increasingly well-accepted that the cell inheriting more fusome material is more likely to become the oocyte, rather than a random selection from the two cells with four ring canals (Lin and Spradling, 1995; Nashchekin et al., 2021). By region 2b, the oocyte has been selected and is the only cell to remain in meiosis. Finally, as the cyst moves down to region 3 of the germarium (also called stage 1 of oogenesis), somatic follicle cells migrate and surround the cyst to form an egg chamber (Jagut et al., 2013). As the cysts pass down the ovariole, they mature into egg chambers containing 15 nurse cells and one oocyte surrounded by follicular epithelium. Nurse cells play a crucial role in supporting oocyte development by providing nutrients and cellular components. *Drosophila* oogenesis is divided into 14 stages in which the progressive morphogenesis of the oocyte and epithelium occurs (Figure 1). A particularly fascinating phase of the egg chamber development occurs during stages 9–10, when collective migration of the border cells is observed (Bastock and St Johnston, 2008; Peercy and Starz-Gaiano, 2020). These highly specialized cells undergo a partial epithelial-to-mesenchymal transition and navigate as a cell cluster between nurse cells towards the developing oocyte. Once oocyte growth is completed, nurse cells rapidly transfer their cytoplasm into the oocyte. Finally, the micropyle is formed in a dorso-anterior region of the egg chamber, adjacent to the oocyte nucleus. This structure enables fertilization, and its formation requires the participation of four types of cells: the anterior polar cells, border cells, proximally located centripetal cells, and the oocyte (Horne-Badovinac, 2020). Migration of border cells is regulated by complex cytoskeletal rearrangements that include the formation of lamellipodia, stabilization of the border cell cluster, and guidance of border cells between nurse cells (Montell et al., 2012). This sophisticated regulation provides a compelling model to study the role of the cytoskeleton in collective cell migration in the context of developmental biology and cancer metastasis (Yoshida et al., 2004; Friedl and Gilmour, 2009; Scarpa and Mayor, 2016).

Myosin VI was first identified in *Drosophila melanogaster* as a protein encoded by the *jaguar*/95F gene (Kellerman and Miller, 1992). This actin-based molecular motor is unique among known myosins for its ability to move towards the minus end of actin filaments (Wells et al., 1999). Myosin VI has been implicated in a variety of cellular functions in *Drosophila*. For example, it is involved in membrane remodeling during embryogenesis (Mermall and Miller, 1995; Deng et al., 1999), in asymmetric protein localization in neuroblasts during cell division (Petritsch et al., 2003), as well as in epithelial cell morphogenesis (Millo et al., 2004). Moreover, complete loss of myosin VI function or lack of this protein only in the testes causes infertility in *Drosophila* males (Hicks et al., 1999; Noguchi et al., 2006; Morrison and Miller, 2008; Zakrzewski et al., 2021). Myosin VI activity in various animals, including mammals, is mediated by several cargo adaptor proteins that form a molecular link between the actin cytoskeleton and fundamental cellular processes such as endocytosis, autophagy, secretion, regulation of actin organization and dynamics, and cell motility (Chibalina et al., 2009; Tumbarello et al., 2013; de Jonge et al., 2019). Myosin VI has also been suggested to play an important role in egg chamber development and border cell migration in *Drosophila* ovary (Deng et al., 1999; Geisbrecht and Montell, 2002; Millo and Bownes, 2007). It should be noted that border cell migration is necessary for the successful completion of oogenesis and micropyle formation, and is therefore essential for fertility (Montell et al., 1992). However, *Drosophila jaguar* ^*322*^ mutant females, characterized by complete myosin VI loss of function, are fertile and produce offspring in numbers equal to control non-mutant animals (Morrison and Miller, 2008). This indicates that border cell migration should function normally in myosin VI-deficient *Drosophila* females. In this work, we show for the first time that myosin VI is expressed in *Drosophila* germarium and in very early ovarian follicles, including the polar cells and developing oocyte. Moreover, we confirm that this protein is also present in the nascent border cell cluster and then when the cluster detaches from the epithelium and begins the migration process. Finally, using transgenic flies and myosin VI-null zygotic mutants, we re-examine the potential role of myosin VI in border cell migration and egg chamber development.

## Materials and methods

### Fly stocks, husbandry, and crosses

**FIGURE 2:**
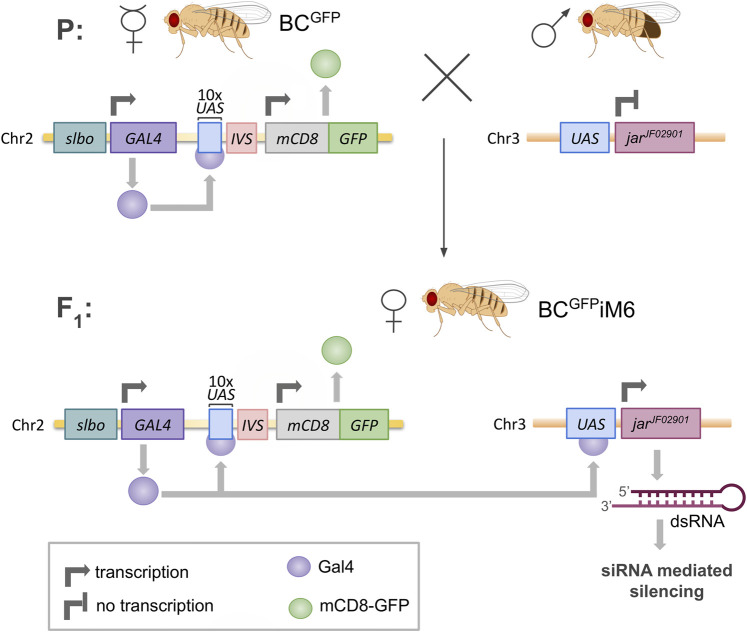
Scheme of silencing of *jaguar* (*jar*) gene expression in *Drosophila* border cells. BC^GFP^ females contain a *GAL4* sequence driven by transcriptional control of a minimal promoter linked to the *slbo* gene enhancer, which is specific for border cells. Gal4 protein acts as a transcription factor, binding to ten repeated upstream activating sequences (*UAS*), thereby enabling expression of *mCD8-GFP* sequence in border cells. The males contain *jar* ^*JF02901*^ sequence preceded by *UAS*, which codes dsRNA for silencing the myosin VI gene (*jar*). The offspring females (BC^GFP^iM6) exhibit *slbo*-Gal4-driven expression of both mCD8-GFP and dsRNA for siRNA-mediated silencing of *jar* in border cells.

The following *D. melanogaster* strains were obtained from the Bloomington *Drosophila* Stock Center (IN, US) and used in the experiments: (1) w[*]; P{w[+mC]=GAL4-slbo.2.6}16, P{y [+t7.7]w[+mC]=10XUAS-IVS-mCD8::GFP}attP40 (Bloomington stock 76363) with two inserted elements: P{10XUAS-IVS-mCD8::GFP} (expresses mCD8-tagged GFP under the control of 10 UAS sequences with an intron - IVS - interposed between the UAS and coding sequences) and P{GAL4-slbo.2.6} (expresses GAL4 in the pattern of slbo), females used as the transgenic flies expressing GFP in border cells (called BC^GFP^ in this paper); (2) y[1]v[1]; P{y[+t7.7]v[+t1.8]=TRiP.JF02901}attP2 (Bloomington stock 28064) with inserted element P{TRiP.JF02901} (expresses dsRNA for RNAi of jar - FBgn0011225 – under UAS control in the VALIUM10 vector), males used for crossing with BC^GFP^ virgin females to obtain offspring with silenced expression of myosin VI in border cells (called BC^GFP^iM6 in this paper). This methodology, shown in Figure 2, is based on the Transgenic RNAi Project at Harvard Medical School (Perkins et al., 2015). In addition, myosin VI-null zygotic animals (called *jar* ^*322*^ or myosin VI mutant in this paper) were generated by crossing *Drosophila* virgin females Df(3R)jar[322], jar[322] beta-PheRS[322]/TM3, P{w [+m*]=Ubx-lacZ.w[+]}TM3, Sb[1] (Bloomington stock 8776) to Df(3R)crb87-5, st[1]e[1]/TM3, Ser[1] (Bloomington stock 2363) males, combination used previously by Morrison and Miller (2008). Oregon R strain (obtained from Kathryn G. Miller, Washington University in St. Louis, MO, US) was used as the wild-type control (called WT in this paper). Flies were raised on standard cornmeal agar medium supplemented with yeast at 25°C and crosses were performed under standard conditions. Adult male and female flies (two- and three-day old) were used in the experiments.

### Egg chambers preparation for stereoscopic and fluorescence microscopy

Newly eclosed flies (females and males) were collected in a fresh bottle with fly food for two or three days. Flies were anesthetized with CO_2_, then whole ovary pairs were dissected from the females in 0.1 M phosphate-buffered saline (PBS), pH 7.0, and immediately transferred to freshly prepared fixative placed on ice. For stereoscopic microscopy (morphological analysis of ovaries), dissected ovary pairs were fixed with 1.5% glutaraldehyde in PBS, pH 7.0, for 15 min at room temperature, then washed with the same phosphate buffer and examined using the Zeiss Discovery V8 stereo microscope and AxioVs40V4.8.2.0 software. For fluorescence microscopy (analysis of border cell migration), dissected ovaries were fixed with 4% formaldehyde in 0.1 M PBS, pH 7.0, for 30 min at room temperature and washed with the same PBS buffer. The samples of separate ovarioles were covered with mounting medium to prolong the GFP fluorescence (ProLong™ Gold antifade reagent, Invitrogen by Thermo Fisher Scientific) and examined using the Nikon Eclipse 80i fluorescence microscope and NIS-Elements AR.3.00 software. Morphological analysis of ovaries and visualization of the border cell cluster formation and migration for each tested genotype were repeated several times and representative data were presented.

### Immunolabeling and confocal microscopy

For immunocytochemical studies, the preparation of egg chambers was carried out as described above. Dissected ovaries were fixed with 4% formaldehyde in 0.1 M PBS, pH 7.0, for 30 min at room temperature, washed twice with the same phosphate buffer, and then permeabilized with 0.1% (m/v) saponin in 0.1 M PBS, pH 7.0, for 20 min at room temperature. Next, the samples were blocked with 1% bovine serum albumin (BSA) in the same PBS buffer supplemented with 0.1% (m/v) saponin and 0.25% BSA for 15 min at room temperature, and then incubated with the primary antibody (monoclonal mouse anti-*Drosophila* myosin VI antibody 3C7 (Kellerman and Miller, 1992) diluted 1:20, rotating overnight at 4°C. Samples were then washed three times with PBS buffer and incubated, with rotation, overnight at 4°C with Alexa Fluor Plus 594 goat anti-mouse IgG secondary antibody (Invitrogen by Thermo Fisher Scientific), diluted 1:100 in 0.1 M PBS, pH 7.0 supplemented with 0.1% (m/v) saponin and 0.25% (m/v) BSA. After washing three times in PBS buffer, DNA was stained with Hoechst 33342 stain solution (Invitrogen by Thermo Fisher Scientific). Finally, ovaries were washed in H_2_O mQ and samples of separate ovarioles were covered with antifade mountant to prolong the fluorescence (ProLong™ Gold antifade reagent, Invitrogen by Thermo Fisher Scientific). A negative control omitting the primary antibody was also performed. Imaging was performed on the Olympus Fluoview FV3000 confocal laser scanning microscope and FV31S-SW software. Immunocytochemical experiments were performed several times, and representative data (selected optical sections) were shown.

To verify the efficiency of myosin VI silencing in border cells, quantitative fluorescence measurements and statistical analysis were performed. For quantitative measurements, immunolabeling of myosin VI was carried out with consistent experimental conditions and concentrations of the primary and secondary antibodies, and the same exposure time was used for all analyzed samples. Three-dimensional optical sections of the border cell clusters were acquired with a 1.0 µm step intervals, from a minimum of 20 comparable egg chambers dissected from BC^GFP^ and BC^GFP^iM6 females. All data were corrected for background autofluorescence as determined by signal intensities in negative controls. For image processing and analysis, the Olympus Fluoview FV3000 confocal laser scanning microscope with FV31S-SW software package and ImageJ (NIH, Bethesda, MD, United States) software were used. The fluorescence intensity was measured per single egg chamber (collection of serial optical sections). PAST three software and Microsoft Excel (Microsoft, Redmond, Washington, DC, United States) were used for statistical analysis, and the statistical significance of data was determined using the Mann-Whitney test.

### Stain-free western blot analysis

To assess the absence of myosin VI in *Drosophila jar* ^*322*^ mutant, stain-free Western blot analysis was performed. WT and myosin VI-null zygotic adults flies were ground in liquid nitrogen, and proteins were extracted using a buffer containing 100 mM Tris-HCl (pH 7.5), 10% sucrose, 5 mM EGTA, 2 mM DTT, and cOmplete Protease Inhibitor Cocktail (Roche). Equal volumes of protein extracts were denatured at 95°C for 5 min, centrifuged, and then run on a 10% TGX stain-free gel (Bio-Rad) at 140 V for 90 min. The electrophoretically separated proteins were fluorescently labeled with a trihalo compound using the ChemiDoc™ Touch Imaging System (Bio-Rad), followed by transfer onto an Immune-Blot LF PVDF Membrane (Bio-Rad). Fluorescence signals of the trihalo-modified proteins were captured with the ChemiDoc™ Touch Imaging System. Subsequently, the membranes were probed with a monoclonal mouse anti-*Drosophila* myosin VI antibody 3C7 (Kellerman and Miller, 1992), washed, and incubated with a horseradish peroxidase-conjugated secondary antibody (Merck). Specific antigens were detected using the Amersham ECL Advance Western blotting Detection Kit (Cytiva) and visualized with the ChemiDoc™ Touch Imaging System.

### Fertility assays

To assess the fertility of BC^GFP^iM6 females, they were mated with WT males and the number of progeny was compared to that obtained after crossing (1) BC^GFP^ females with WT males and (2) WT females with WT males. Twenty virgin adult females of the test genotype (WT, BC^GFP^, and BC^GFP^iM6) were placed with twenty adult WT males (1–2 days after eclosion) in a small vial with fly food at 25°C. The next day, adults were transferred to a bottle with fresh fly food (day 0, 25°C) and then removed 7 days later. Progeny in each bottle was counted until day 18. The number of bottles counted was 10 for each genotype tested and the average number of progeny per bottle was reported. To assess the fertility of myosin VI-null zygotic females (*jar* ^*322*^/Df (3R) S87-5), they were mated with WT males and the number of progeny was compared to that obtained after crossing control females (*jar* ^*322*^/TM3 Sb) with WT males. In this case, three virgin adult females were crossed with three WT adult males. The number of bottles counted was five for each genotype tested and the average number of progeny per bottle was reported.

## Results

### Myosin VI is present in ovarian follicles during the early steps of *Drosophila* oogenesis

**FIGURE 3:**
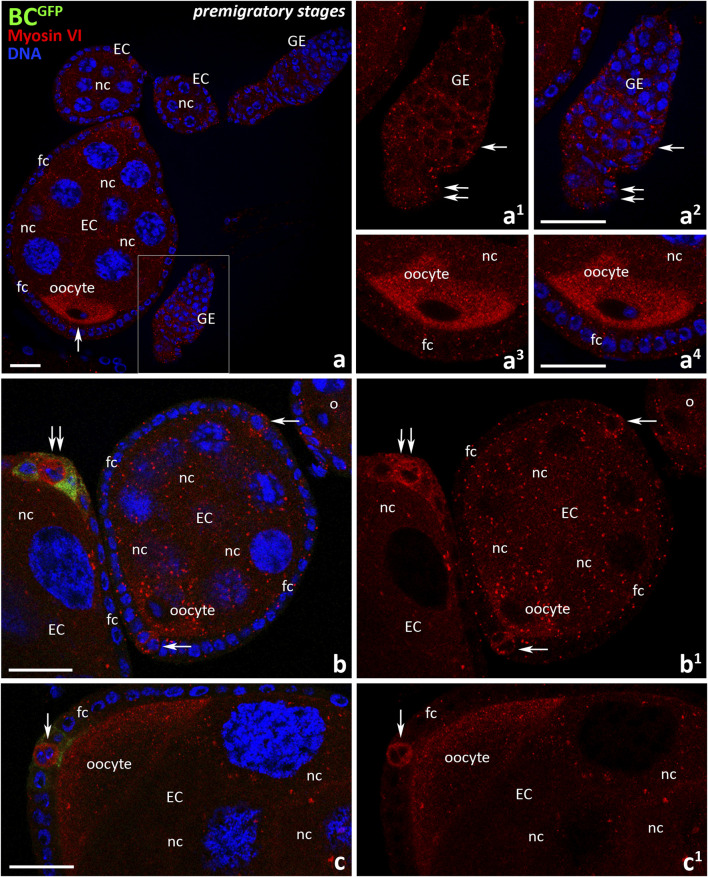
Immunocytochemical localization of myosin VI during the early (premigratory) stages of oogenesis in *Drosophila* control BC^GFP^ females. Images show: germarium and early ovarian follicles before the specification/recruitment of border cells **(a)**, germarium **(a** ^**1**^ **, a** ^**2**^ **)**, developing oocyte at stage 7 **(a** ^**3**^ **, a** ^**4**^ **)**, early ovarian follicle at stage 6 and anterior pole of the egg chamber at stage 8 **(b, b** ^**1**^ **)**, posterior pole of the egg chamber at stage 8 **(c, c** ^**1**^ **)**. The region marked in **(a)** is shown in a larger magnification in **(a** ^**1**^ **, a** ^**2**^ **)**. Recruiting border cells are stained in green, myosin VI is stained in red, and cell nuclei are stained in blue. Arrows point respectively: oocyte **(a)**, region 2a/b of the germarium **(a** ^**1**^ **, a** ^**2**^ **)**, polar cells **(b-c** ^**1**^ **)**; double arrows point respectively: region 2a/b of the germarium **(a** ^**1**^ **, a** ^**2**^ **)**, polar cells at the border cell recruitment **(b, b** ^**1**^ **)**. EC, egg chamber; GE, germarium; bc, border cells; fc, follicle cells; nc, nurse cells. Bars 25 μm.

Since the presence and localization of myosin VI in *Drosophila* ovarian follicles at early stages of oogenesis (also called premigratory stages) has not been previously documented, we performed a series of immunocytochemical experiments using anti-myosin VI antibody and confocal microscopy in BC^GFP^ control females. In the first step, we investigated the localization of myosin VI in the germarium and egg chambers up to stage 8 of oogenesis (Figure 1). As shown in Figure 3, myosin VI was present in both germarium and early ovarian follicles in which specification/recruitment of border cells has not yet occurred, with a particular accumulation of this protein found in the developing oocyte (Figure 3a, arrow). In the germarium, myosin VI was present in all segments, including region 2a/b (Figures 3a^1^, a^2^, arrows) and posterior region 3 (Figures 3a^1^, a^2^, double arrows). In the growing oocyte, we observed increasing accumulation of myosin VI throughout the oocyte cytoplasm (Figures 3b, b^1^, Figures 3a^3^, a^4^) until stage 8 of oogenesis, when the localization pattern changed and the immunofluorescence signal became dominant at the posterior pole of the oocyte (Figures 3c, c^1^). Myosin VI was also present in the supporting nurse cells and epithelial cells surrounding developing egg chambers (Figures 3a-c^1^). In nurse cells, a punctate distribution of myosin VI in their cytoplasm was observed (Figures 3b, b^1^). Among the follicular epithelial cells, particularly localization of myosin VI was found in polar cells located both at the anterior and posterior poles of early egg chambers up to stage 8 of oogenesis (Figures 3b-c^1^ arrows). At stage 8, GFP fluorescence appeared, corresponding to the specification/recruitment of border cells (Figures 3b, b^1^, double arrows). A negative control omitting the primary antibody was also performed, which showed a complete lack of nonspecific red fluorescence in early ovarian follicles of BC^GFP^ females (Supplementary Figure S1).

**FIGURE 4:**
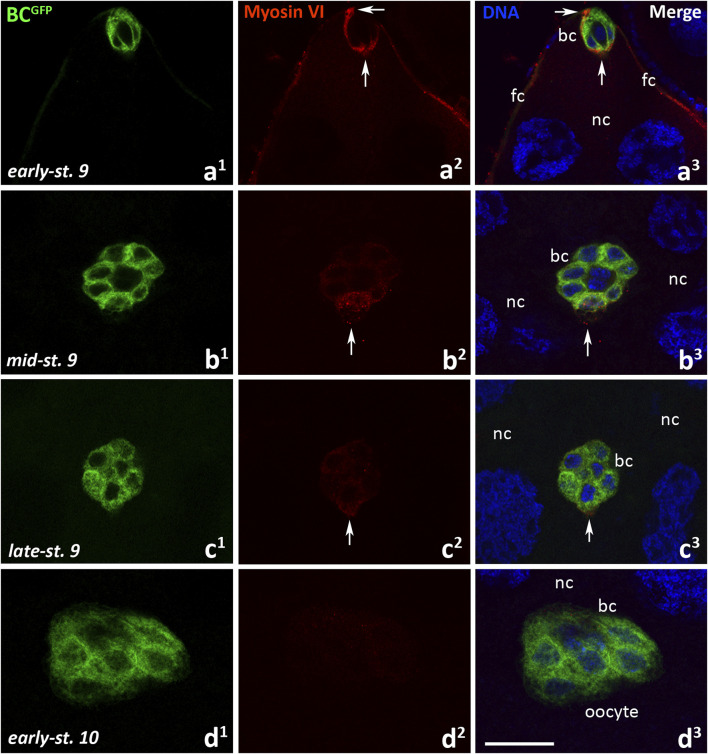
Immunocytochemical localization of myosin VI in the egg chambers of control BC^GFP^ *Drosophila* females during border cell migration: the early-stage 9, start of border cell migration **(a** ^**1**^ **-a** ^**3**^ **)**, mid-stage 9, migration of border cells between nurse cells **(b** ^**1**^ **-b** ^**3**^ **)**, late-stage 9, continuation of border cell migration **(c** ^**1**^ **-c** ^**3**^ **)**, and early-stage 10, border cells reach the oocyte **(d** ^**1**^ **-d** ^**3**^ **)**. Border cells are stained in green, myosin VI is stained in red, and cell nuclei are stained in blue. Arrows show respectively: the front and rear of the border cell cluster **(a** ^**2**^ **, a** ^**3**^ **)**, the leader edge/cell of the border cell cluster **(b** ^**2**^ **, b** ^**3**^ **, c** ^**2**^ **, c** ^**3**^ **)**. bc, border cells; fc, follicular cells; nc, nurse cells. Bar 25 µm.

We next examined the localization of myosin VI during border cell migration in *Drosophila* BC^GFP^ control females. As shown in Figure 4, the successive migratory stages of oogenesis were examined: delamination of the border cell cluster from the epithelium–start of migration (early-stage 9, Figures 4a^1^-a^3^), migration of border cells between nurse cells (mid-stage 9, Figures 4b^1^-b^3^), continuation of border cell migration towards the oocyte (late-stage 9, Figures 4c^1^-c^3^), border cells reaching the oocyte (early-stage 10, Figures 4d^1^-d^3^). When the border cell cluster delaminated and started to move (Figures 4a^1^-a^3^), a strong myosin VI fluorescence signal was present around the cluster and the protein accumulated both at its leading edge and at the point of detachment of the cluster from the epithelium (Figures 4a^2^, a^3^, arrows; Supplementary Figures S2a, a^1^, arrows). At this stage, myosin VI signal was also visible in the follicular epithelium (Figures 4a^2^, a^3^; Supplementary Figure S2a, arrow heads). By the mid-stage 9, when border cells migrate between nurse cells towards the oocyte (Figures 4b^1^-b^3^), immunolabeling of myosin VI in the border cell cluster was particularly associated with the leader cell of the cluster (Figures 4b^2^, b^3^, arrows). However, myosin VI signal in the leader cell disappeared as border cells continued their migration towards the oocyte (Figures 4c^2^, c^3^, arrows), and was no longer detected in the early-stage 10 as the cluster reached the oocyte (Figures 4d^1^-d^3^). Myosin VI was consistently detectable (with varying intensity) in the follicular epithelium surrounding the anterior (Supplementary Figures S2b-d, arrow heads) and posterior (Supplementary Figures S2c, d, double arrow heads) regions of the egg chamber. A negative control omitting the primary antibody was also performed and showed a complete lack of nonspecific red fluorescence in the egg chamber of BC^GFP^ females (Supplementary Figure S2e). These results indicate that myosin VI is expressed in *Drosophila* germarium as well as in early ovarian follicles up to step 8 of oogenesis, especially in the developing oocyte and polar cells. However, as oogenesis progresses, the myosin VI signal in egg chambers decreases, but is present in the nascent border cell cluster and during its early/middle (but not late) stages of migration.

### Border cell migration is effective in *Drosophila* females with silenced expression of myosin VI in the cluster

In the context of previous reports indicating an important role of myosin VI in border cell migration in *Drosophila* (Geisbrecht and Montell, 2002), we expected to observe high level of its expression in migrating cluster from the stage 9 to the stage 10 of oogenesis. However, our results (Figure 4; Supplementary Figure S2) did not confirm these observations. We therefore wanted to re-examine the role of myosin VI in border cell migration and, consequently, the progression of oogenesis. As a result of the cross between appropriate parental individuals we obtained BC^GFP^iM6 females, in which the border cells express GFP and myosin VI expression silenced (Figure 2).

**FIGURE 5:**
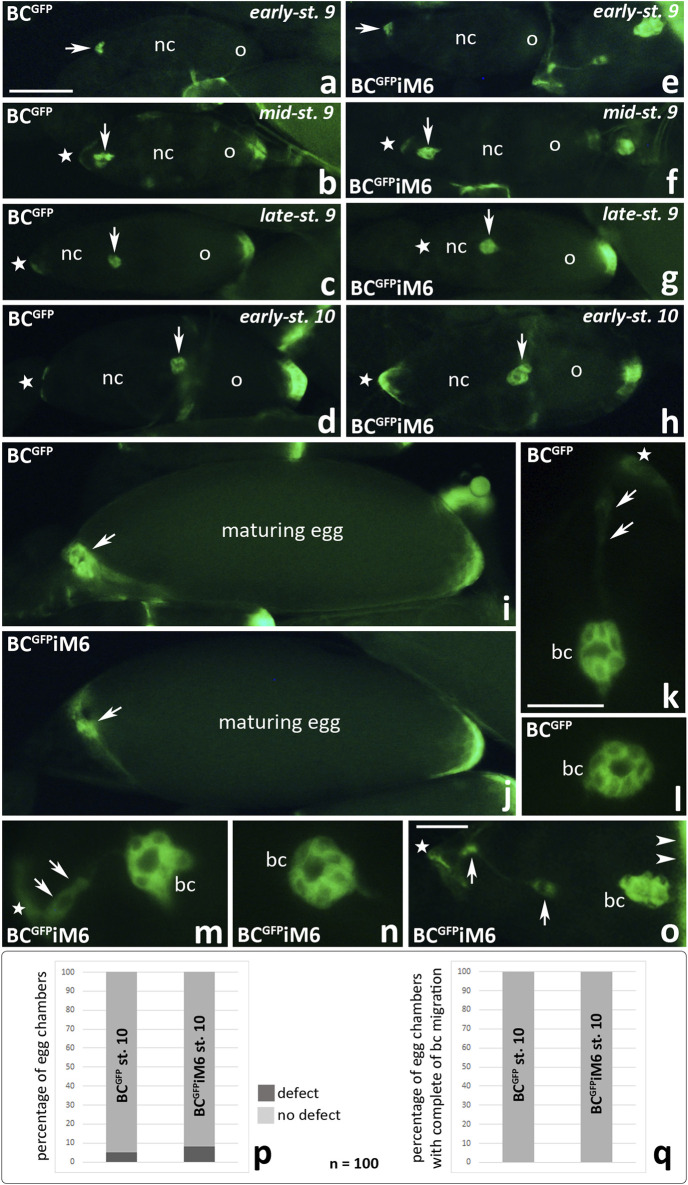
Fluorescence analysis of border cell migration in the egg chambers of *Drosophila* BC^GFP^iM6 and control BC^GFP^ females during subsequent stages of oogenesis: start of migration **(a, e)**, migration of border cells **(b, f, c, g, k-n)**, border cells reach the oocyte **(d, h, o)**. Arrows show respectively: the border cell cluster **(a–h)**, the anterior pole of maturing egg **(i, j)**, a trace of green fluorescence between the migrating cluster and the epithelium in the control egg chamber **(k)**, and incomplete separation of the border cell cluster from the epithelium in the egg chamber of BC^GFP^iM6 females **(m, o)**; arrow heads show the anterior pole of the oocyte **(o)**; stars show the anterior pole of the egg chambers **(b-h, k, m, o)**. The graphs **(p)** show the number of egg chambers with a border cell cluster integrity defect (a trace of green fluorescence behind the cluster of border cells that have reached the oocyte) per 100 egg chambers at stage 10 of oogenesis in BC^GFP^iM6 and control females. The graphs **(q)** show that 100% of the border cell clusters reached the oocyte at stage 10 of oogenesis in both BC ^GFP^iM6 (n = 100) and control (n = 100) females. bc, border cells; nc, nurse cells; o, oocyte. Bars 10 µm **(a–j)**, 5 µm **(k–o)**.

To test whether myosin VI deficiency in border cells inhibits their migration, we first analyzed individual egg chambers of BC^GFP^iM6 and BC^GFP^ control females to visualize migrating border cell clusters under a fluorescence microscope. As shown in Figure 5, no significant defects in border cell migration were observed in BC^GFP^iM6 females, as compared to control females. For both tested genotypes, border cell clusters (Figures 5a–h, arrows) remained clearly visible throughout successive stages of oogenesis: emergence/delamination of the border cell cluster at the anterior pole of the egg chamber (Figures 5a, e), posterior migration of border cells between nurse cells (Figures 5b, c, f, g), and upon reaching the oocyte (Figures 5d, h). In both cases (BC^GFP^iM6 and BC^GFP^ control females) maturing eggs were present in the ovaries with border cells located at their anterior pole (Figures 5i, j, arrows), after nurse cells completed dumping process. These results indicate that myosin VI deficiency in border cells did not affect progression of oogenesis. Higher magnifications of the egg chambers at stage 9 of oogenesis illustrate the migrating border cell clusters that contain two non-migratory polar cells (Figures 5k–n). We found that in BC^GFP^ control females, the border cell cluster was usually perfectly formed (Figures 5k, l). Occasionally we observed a trace of green fluorescence between the migrating cluster and the epithelium at the anterior pole of the egg chamber (Figure 5k, arrows). In BC^GFP^iM6 females, the migrating border cell cluster was also correctly formed (Figures 5m, n). However, silencing of myosin VI expression in border cells resulted in incomplete separation of the migrating cluster from the epithelium in some cases (Figures 5m, o, arrows) or partial disruption of the cluster integrity even the cluster moved between nurse cells to reach the oocyte at early-stage 10 (Figure 5o, arrow heads). As shown in Figure 5p, these defects occurred in less than 5% of egg chambers in BC^GFP^ control females (5 per 100 egg chambers at stage 10 of oogenesis) and in approximately 10% of egg chambers in BC^GFP^iM6 females (8 per 100 egg chambers at stage 10 of oogenesis). However, in every case of the 100 analyzed egg chambers at stage 10 of oogenesis (BC^GFP^iM6 and control females), the border cell cluster reached the oocyte (Figure 5q). We do not know whether this subtle defect had any negative consequences on the formation of mature eggs, but since we did not observe the border cell clusters between nurse cells at stage 10 of oogenesis, we conclude that border cell migration and egg development is effective in myosin VI-deficient *Drosophila* females.

**FIGURE 6:**
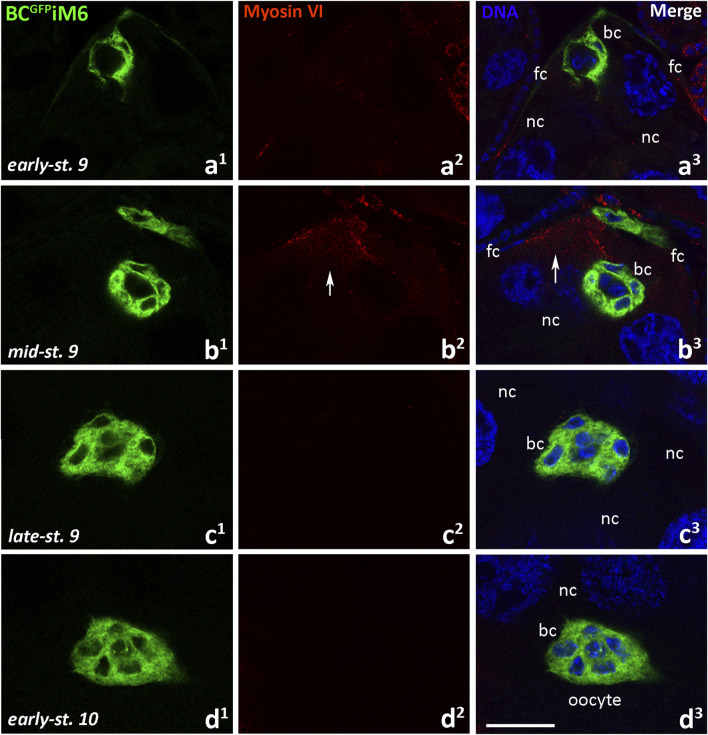
Immunocytochemical localization of myosin VI in the egg chambers of BC^GFP^iM6 *Drosophila* females (depleted of myosin VI in border cells) during border cell migration: the early-stage 9, start of border cell migration **(a** ^**1**^ **-a** ^**3**^ **)**, mid-stage 9, migration of border cells between nurse cells **(b** ^**1**^ **-b** ^**3**^ **)**, late-stage 9, continuation of the border cell migration **(c** ^**1**^ **-c** ^**3**^ **)**, and early-stage 10, border cells reach the oocyte **(d** ^**1**^ **-d** ^**3**^ **)**. Border cells are stained in green, myosin VI is stained in red, and cell nuclei are stained in blue. Arrows show the labeling in nurse cells. bc, border cells; fc, follicular cells; nc, nurse cells. Bar 25 µm.

We then examined localization of myosin VI in egg chambers dissected from BC^GFP^iM6 females using confocal microscopy (Figure 6). As expected, we did not detect myosin VI in border cells when they started migration towards the oocyte (Figures 6a^1^-a^3^), in migrating clusters (Figures 6b^1^-c^3^) or when the clusters reached the oocyte (Figures 6d^1^-d^3^). However, myosin VI signal was detectable in nurse cells (Figures 6b^2^, b^3^, arrows; Supplementary Figures S3b^2^, c, d, arrows) as well as in the follicular epithelium (Supplementary Figures S3b^1^, d, e, arrow heads), demonstrating that RNAi was limited to the border cells. To verify the efficiency of myosin VI silencing in border cells, we performed a statistical analysis of myosin VI immunofluorescence in the egg chambers dissected from BC^GFP^iM6 and BC^GFP^ control females. This analysis was performed for early-stage 9 of oogenesis, when the cluster started migration and the protein level was highest in border cells. Our quantitative analysis confirmed that the level of myosin VI was about 90% lower in border cells of BC^GFP^iM6 females compared to control females (Supplementary Figure S3a). A negative control omitting the primary antibody was also performed and showed a complete lack of nonspecific red fluorescence in the egg chamber of BC^GFP^iM6 females (Supplementary Figure S3f). Together, we conclude that deficiency of myosin VI in border cells does not affect the border cell migration process, which occurs efficiently.

### Myosin VI deficiency does not affect ovarian morphology and female fertility in *Drosophila*

**FIGURE 7:**
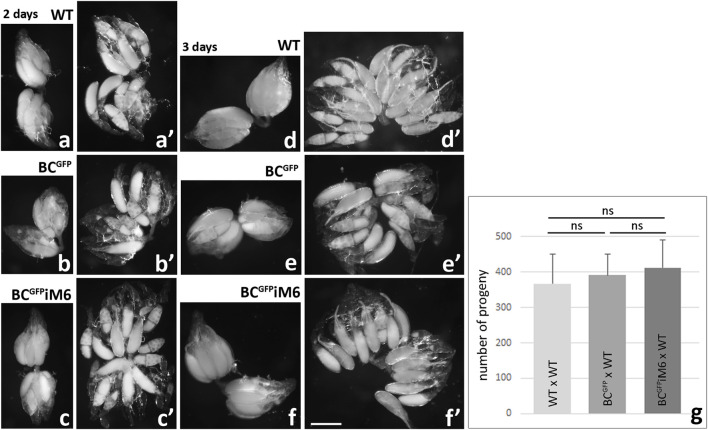
Morphological analysis of *Drosophila* ovaries dissected from WT, BC^GFP^ and BC^GFP^iM6 females and fertility test. Ovaries dissected from 2-day old and 3-day old control (WT, BC^GFP^) and BC^GFP^iM6 females are similar at the same stage of development. Images show whole ovaries **(a-c, d-f)** and gently crushed preparations of fixed ovaries **(a’-c’, d’-f’)**. Ovaries dissected form 2-day-old WT, BC^GFP^iM6 and BC^GFP^iM6 females possess ovarioles composed of egg chambers at various stages of development with a predominance of those at stage 10 of oogenesis, and 3-day-old females possess ovarioles containing mature eggs. Bar 50 µm. Fertility tests of *Drosophila* control (WT and BC^GFP^) and BC^GFP^iM6 females show similar results for all tested genotypes (**g**). Graphs show the number of offspring resulting from the BC^GFP^iM6 virgin females mating with WT males in comparison to the number of offspring obtained from crosses of BC^GFP^ virgin females with WT males or of WT virgin females with WT males (mean of 10 replicates/bottles for each test genotype/offspring and standard deviation; ns, not significant). Statistical analysis was carried out by the one-way ANOVA (***p ≤ 0.001).

Next we decided to compare the morphology of ovaries dissected from BC^GFP^iM6 females and control females (BC^GFP^ and WT). As shown in Figure 7, two-day-old WT females possess ovarioles composed of egg chambers at various stages of development with a predominance of those at stage 10 of oogenesis (Figures 7a, a’), where the size of the oocyte corresponds to approximately half size of the egg chamber (Figure 1). The ovaries appeared similar in two-day-old BC^GFP^ (Figures 7b, b’) and BC^GFP^iM6 (Figures 7c, c’) females. A comparative analysis of ovarian morphology was also performed in three-day-old females, in which the progressive development of ovaries towards mature eggs was observed (Figures 7d-f’). In all *Drosophila* females representing different genotypes, the ovaries contained ovarioles at various stages of development, with a predominance of maturing and mature eggs. Together, we conclude that myosin VI deficiency in border cells does not impact morphology of ovary and the egg chamber development. In addition, to determine the potential impact of myosin VI silencing in border cells on *Drosophila* oogenesis and/or the formation of viable eggs capable of fertilization, we assessed the fertility of BC^GFP^iM6 females compared to control females. Fertility assay did not reveal any significant differences, as the number of offspring resulting from the BC^GFP^iM6 virgin adult females mating with WT adult males in comparison to the number of offspring obtained from crosses of BC^GFP^ virgin adult females with WT adult males or of WT virgin adult females with WT adult males (Figure 7g). Thus, we conclude that silencing of myosin VI expression in border cells did not affect the fertility of *Drosophila* females.

**FIGURE 8:**
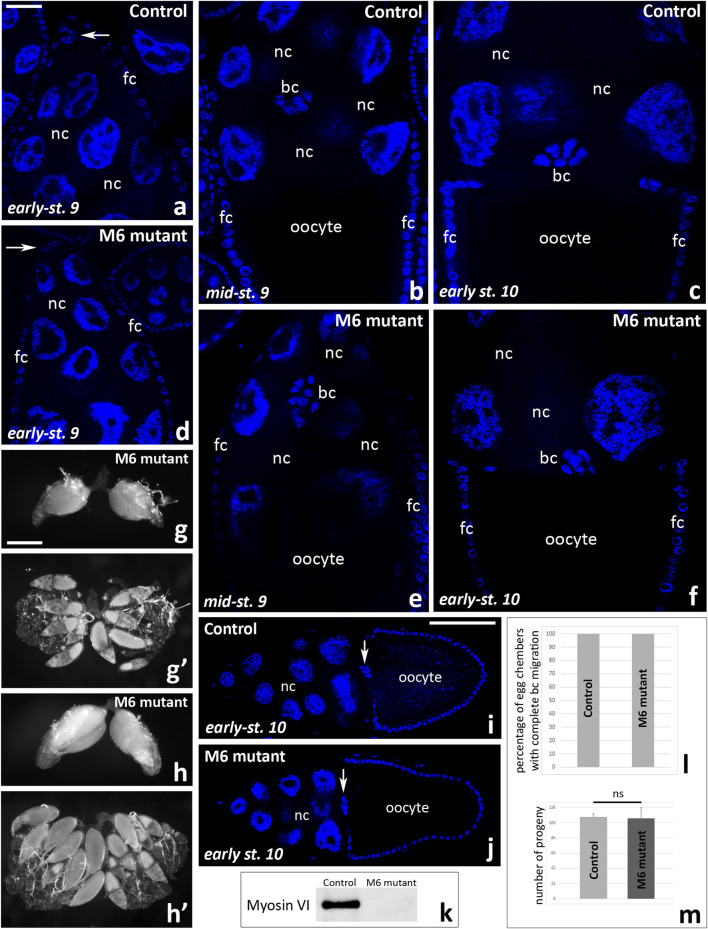
Border cell migration and ovarian morphology in *Drosophila* myosin VI-null zygotic females and control females. A comparative analysis of the egg chambers using fixed and Hoechst 33342-stained ovaries show that border cell migration proceeds effectively in both control (*jar* ^*322*^/TM3 Sb, **a**-**c**) and myosin VI mutant (*jar* ^*322*^/Df (3R) S87-5, **(d-f)** females. Ovarian morphology is also normal; ovaries dissected form two-day-old myosin VI mutant females possess ovarioles composed of egg chambers at various stages of development with a predominance of those at stage 10 of oogenesis **(g, g’)**, and three-day-old females possess ovarioles containing mature eggs **(h, h’)**. Quantitative analysis of egg chambers at stage 10 of oogenesis at n = 50 for each analyzed genotype **(i, j)** showed that 100% of border cell clusters reached the oocyte **(l)**. Fertility tests of *Drosophila* control and myosin VI mutant females show similar results for both tested genotypes **(m)**. Graphs show the number of offspring resulting from the *jar* ^*322*^/TM3 Sb virgin females mating with WT males in comparison to the number of offspring obtained from crosses of *jar* ^*322*^/Df (3R) S87-5 virgin females with WT males (mean of five replicates/bottles for each test genotype/offspring and standard deviation; ns, not significant). Statistical analysis was carried out by the one-way ANOVA (***p ≤ 0.001). Immunoblot shows no detection of myosin VI in *jar* ^*322*^/Df (3R) S87-5 females **(k)**. Arrows in **(a, d, i, j)** show the border cell cluster. bc, border cells; fc, follicular cells; nc, nurse cells. Bars 25 µm **(a–f)**, 50 µm **(g-h’)**, 100 µm **(i, j)**.

Finally, to test whether complete lack of myosin VI inhibits border cell migration, we performed control experiments according to the combination proposed previously (Morrison and Miller, 2008). We dissected ovaries from myosin VI-null zygotic females (*jar* ^*322*^ mutant), fixed them with formaldehyde, stained the nuclei, and analyzed the border cell migration process using confocal microscopy. This simple staining proved sufficient to visualize the cluster of border cells at different stages of oogenesis both in whole egg chambers (Figures 8i, j) and at higher magnification, showing the typical shape of the cluster (Figures 8a–f). As shown in Figure 8, border cell migration proceeds effectively in both control females (*jar* ^*322*^/TM3 Sb, Figures 8a–c) and myosin VI mutant females (*jar* ^*322*^/Df (3R) S87-5, Figures 8d–f). Moreover, the ovarian morphology is normal in myosin VI-null zygotic females; two-day-old females possess ovarioles composed of egg chambers at various stages of development with a predominance of those at stage 10 of oogenesis (Figures 8g, g’), and three-day-old females possess ovarioles containing mature eggs (Figures 8h, h’). We also performed quantitative analysis of egg chambers at stage 10 of oogenesis in control females (Figure 8i) and myosin VI mutant females (Figure 8j) and showed that 100% of border cell clusters reached the oocyte in each genotype tested (Figure 8l). Finally, fertility tests of *Drosophila* control and myosin VI mutant females showed similar results (Figure 8m) and immunoblot confirmed that no myosin VI was detected in *jar* ^*322*^/Df (3R) S87-5 flies (Figure 8k). Taken together, our present studies show that complete loss of function of myosin VI does not impair border cell migration and the egg chamber development during oogenesis in *Drosophila*. Moreover, we confirmed the previous results by Morrison and Miller (2008) that myosin VI-null zygotic females are fertile and, compared to control genotypes, produce offspring in numbers equal to non-mutant animals.

## Discussion

### Myosin VI is expressed during the early steps of *Drosophila* oogenesis

*Drosophila melanogaster* is a particularly well-studied model of oogenesis, and numerous genes and pathways that are crucial for the specification and differentiation of a viable female gametes have been identified (Cabrita and Martinho, 2023). However, despite the undisputed role of the actin cytoskeleton and various actin-binding proteins in fruit fly oogenesis, the potential involvement of actin-dependent motor proteins in this process is still a matter of debate. In fact, the results obtained over the years indicate that some myosins may have important roles in *Drosophila* oogenesis. For example, *Drosophila* nonmuscle myosin II has been shown to be involved in the egg chamber morphogenesis, oogenesis and early embryogenesis (Wheatley et al., 1995; Edwards and Kiehart, 1996; Wang and Riechmann, 2007). Myosin II has been also implicated in rapid cytoplasmic transport during oogenesis (nurse cell dumping) and axial nuclear migration in early embryos (Wheatley et al., 1995; Edwards and Kiehart, 1996). Moreover, a recent elegant study by Doerflinger et al. (2022) showed that anterior-posterior polarization of the *Drosophila* oocyte at mid-oogenesis (stages 7–9) requires nonmuscle myosin II. It is also well established that myosin V participates in local accumulation of *oskar* mRNA and Staufen particles at the posterior pole of the *Drosophila* oocyte (Krauss et al., 2009; Lu et al., 2020). Despite the fact that myosin V is ubiquitously expressed throughout *Drosophila* development, myosin V mutants did not show any detectable defects during either oogenesis or embryogenesis (Mermall et al., 2005). However, myosin V is strictly required for larval development demonstrating that it is essential in *Drosophila* life cycle. Another myosin that is abundant in developing *Drosophila* ovarian follicles, both in the germline and in the somatic cells of the ovary is myosin VIIA (Glowinski et al., 2014). Subcellular enrichment of this molecular motor showed a strong association with areas rich in actin bundles and actin-rich cellular protrusions, and phenotypic analysis suggests that myosin VIIA is involved in regulating the structure and arrangement of follicle cell and oocyte microvilli as well as in cell migration (Glowinski et al., 2014). Finally, the involvement of myosin VI in *Drosophila* oogenesis has long been suggested, including possible involvement in intra/intercellular transport during mid-oogenesis, morphogenesis of epithelial cells, and cell migration during oogenesis and embryogenesis (Mermall et al., 1994; Mermall and Miller, 1995; Bohrmann, 1997; Deng et al., 1999; Geisbrecht and Montell, 2002; Millo et al., 2004; Millo and Bownes, 2007). Most of these data indicate the involvement of several unconventional myosins, including myosin VI, in later than early stages of oogenesis and embryogenesis of *Drosophila*.

In this paper, we demonstrate that myosin VI is present in *Drosophila* germarium (including regions 2a/b and 3), as well as in early ovarian follicles, particularly in polar cells (at the anterior and posterior poles of the egg chamber) and in the developing oocyte. Interestingly, myosin VIIA has also been shown to localize in *Drosophila* germarium (Glowinski et al., 2014). These authors suggest that the localization pattern confirms accumulation of myosin VIIA in the ring canals–cytoplasmic bridges connecting germline cyst cells. We do not rule out this possibility for myosin VI, as this protein is associated with the ring canals in the germarium of bees suggesting its role in the organization of intracellular transport (Patrício et al., 2010). The role of myosin VI may be also important in the establishment of the egg chamber polarity, including developing oocyte. In wild-type oocytes, the Staufen protein was shown to accumulate in the posterior part of the oocyte until stage 6 and became highly concentrated in the oocyte center at stages 7–8 (Tanaka and Nakamura, 2008). Staufen was then transported to the posterior pole of the oocyte until the completion of oogenesis. Similar dynamics were confirmed in the distribution of several other molecules important in cell polarity during *Drosophila* development (Milas and Telley, 2022). Interestingly, we observed increasing accumulation of myosin VI through the cytoplasm of the growing oocyte until stage 8 of oogenesis, when the localization pattern changed and the immunofluorescence signal became dominant at the posterior pole of the oocyte. These results therefore indicate that the interplay of polarization and signaling pathways in the differentiation of germline and associated somatic cells during oogenesis in *Drosophila* appears to involve myosin VI. However, deficiency of myosin VI in border cells (present work) or even complete absence of this protein in the *Drosophila* myosin VI mutant (present work; Morrison and Miller, 2008) does not significantly disrupt oogenesis, because these females are fertile. We therefore believe that the involvement of myosin VI in the specification and development of the female germline in *Drosophila* is not strictly required, and functional compensation between different myosins is more probable. Further research is needed to verify the presented hypotheses.

### Myosin VI is not essential in border cell migration and egg chamber development

Using a Gal4-UAS targeted expression system combined with antisense RNA, which allows for disruption of function in specific groups of cells, myosin VI has been implicated in border cell migration during *Drosophila* oogenesis (Deng et al., 1999; Geisbrecht and Montell, 2002). These previous experiments demonstrated that myosin VI is recruited to the nascent border cell cluster and is highly expressed during migration until stage 10 of oogenesis. At the start of border cell migration in the myosin VI anti-sense RNA-expressing border cells, these protrusions were lost and border cell movement ceased. In these migrating cells, myosin VI was isolated in a complex with the adhesion proteins E-catherin and *β*-catenin (Geisbrecht and Montell, 2002). Based on these data, myosin VI binding to these adhesion complexes in the plasma membrane could help develop a protrusive force by pushing actin filaments towards their minus ends away from the cell membrane.

Our present research confirms some results of these previous studies. We validated the presence of myosin VI in the emerging border cell cluster, its accumulation in the leading edge protrusions at the initiation of migration, and its localization in the leading cell of the cluster during the early stages of migration. It should be noted that the established model of collective migration of border cells is based on a leader cell at the front of the cluster displaying extensive protrusive behavior (Scarpa and Mayor, 2016; Olson and Nechiporuk, 2018). However, as border cell migration progressed, the myosin VI signal in the migrating cluster disappeared and was not detected when the border cells reached the oocyte. Moreover, myosin VI deficiency in border cells had no inhibiting effect on their migration and associated progression of oogenesis, as we showed using a targeted Gal4-UAS expression system combined with RNAi. We have also demonstrated for the first time that complete lack of myosin VI in myosin VI-null zygotic females does not inhibit border cell migration. Moreover, *Drosophila* females with complete loss of function of myosin VI are fertile and, compared to control genotypes, produce offspring in numbers equal to non-mutant animals (present work; Morrison and Miller, 2008). It is unclear why antisense expression caused defects (as shown by Geisbrecht and Montell, 2002) when none are detectable in null mutant females or using RNAi-based silencing, but one explanation may be off-target effect of the antisense during the previous experiments. Consequently, we suggest that myosin VI may be rather involved in assembling specific complexes of molecular factors necessary for the specification and formation of the border cell cluster and then initiation of migration, but this protein is not strictly required for these events during oogenesis.

In *Drosophila*, myosin VI plays a pivotal role during the last step of spermatogenesis called spermatid individualization (Hicks et al., 1999; Noguchi et al., 2006). This process is driven by long-lived actin cones, which accumulate myosin VI at their fronts. In myosin VI mutants, the structure of actin cones is disrupted and males are sterile. We demonstrated that myosin VI plays an anchoring role during spermatid individualization by tethering different cargo/membranes to actin filaments. Interestingly, the correct targeting and function of myosin VI at the front of actin cones require the conserved RRL motif in myosin VI tail, responsible for binding molecular partners such as GIPC1 (Isaji et al., 2011). Moreover, our recent studies in mice have demonstrated that myosin VI and its selected binding partners are important to maintain the actin-dependent integrity of highly specialized tubulobulbar complexes required for endocytosis during spermiogenesis and spermiation (Zakrzewski et al., 2020). Our studies further indicated that the loss of myosin VI caused disorganization of the tubulobulbar complexes and reduced fertility in male mice. In this context, silencing of myosin VI expression in border cells occasionally resulted in incomplete separation of the migrating cluster from the epithelium, suggesting partial disruption of the cluster integrity. Therefore, myosin VI may stabilize a functional complex of molecular factors necessary for the proper formation of the border cell cluster capable of actin-dependent migration. In migrating *Drosophila* border cells, myosin VI binds and stabilizes E-cadherin and *β*-catenin complexes crucial for cell migration (Geisbrecht and Montell, 2002). Moreover, during early *Drosophila* embryogenesis, this protein plays a role in epithelial morphogenesis (Millo et al., 2004). Therefore, an anchoring role of myosin VI in the formation and stabilization of the border cell cluster is possible.

Myosin VI may cooperate with other myosins in these processes, and functional compensation among the different myosins is possible. Some results from other authors seem to support this idea. For example, the amount of myosin VIIA increases in the germline during follicle formation in the germarium and again at mid-oogenesis (stage 9) and the protein is prominent until late oogenesis (Glowinski et al., 2014). Compared with the germline, somatic expression of myosin VIIA was relatively low in early stages but increased after stage 8 when follicle cells undergo morphogenetic changes. In addition, migrating border cells show high amounts of myosin VIIA in their actin-rich cellular protrusions. On the other hand, nonmuscle myosin II depletion disrupts egg chamber structure and cell migration of three distinct follicle cell populations: the border cells and centripetal cells, and later the dorsal appendage cells (Edwards and Kiehart, 1996). We therefore hypothesize that the lack of myosin VI might be compensated by other motor proteins if the direction of their movement is not crucial in this process. One of the best candidates seems to be nonmuscle myosin II. It has been recently shown that this protein regulates two essential features of border cell migration: the initial detachment of the border cell cluster from the epithelium and the dynamics of cellular protrusions (Majumder et al., 2012; Mishra et al., 2019).

Taken together, we believe that myosin VI is not essential for efficient oogenesis in *Drosophila*, and several observations support this conclusion. First, our results demonstrate that myosin VI deficiency in border cells does not impact morphology and development of ovaries and egg chambers. Second, no significant defects in border cell migration are observed after silencing of myosin VI expression in border cells compared to control females. Third, the fertility assay revealed no significant differences, as the number of offspring resulting from the BC^GFP^iM6 virgin females mated with WT males is comparable to that obtained from control virgin females mated with WT males. Consequently, BC^GFP^iM6 females produce functional eggs and are fully fertile. Finally, ovarian morphology, egg chamber development and border cell migration occur normally in myosin VI-null zygotic females. These females, characterized by complete myosin VI loss of function, are also fertile and produce offspring in numbers equal to control non-mutant animals. In conclusion, our present work resolved one important issue: myosin VI is not a required player in border cell migration in *Drosophila* ovary. However, given the possibility of functional compensation, further investigations are required to elucidate a more subtle mechanism of action of myosin VI in collective migration of border cells.

## Data Availability

The original contributions presented in the study are included in the article/Supplementary Material, further inquiries can be directed to the corresponding author.
