# Supplementary material for: Myosin VI is expressed in developing ovarian follicles in Drosophila but is not essential for effective oogenesis
Source: Front Cell Dev Biol. 2025 Jun 2;13:1535117. doi: 10.3389/fcell.2025.1535117 (PMC12171261; doi:10.3389/fcell.2025.1535117)
Supplement: Supplementary file 1 [file Image2.pdf]

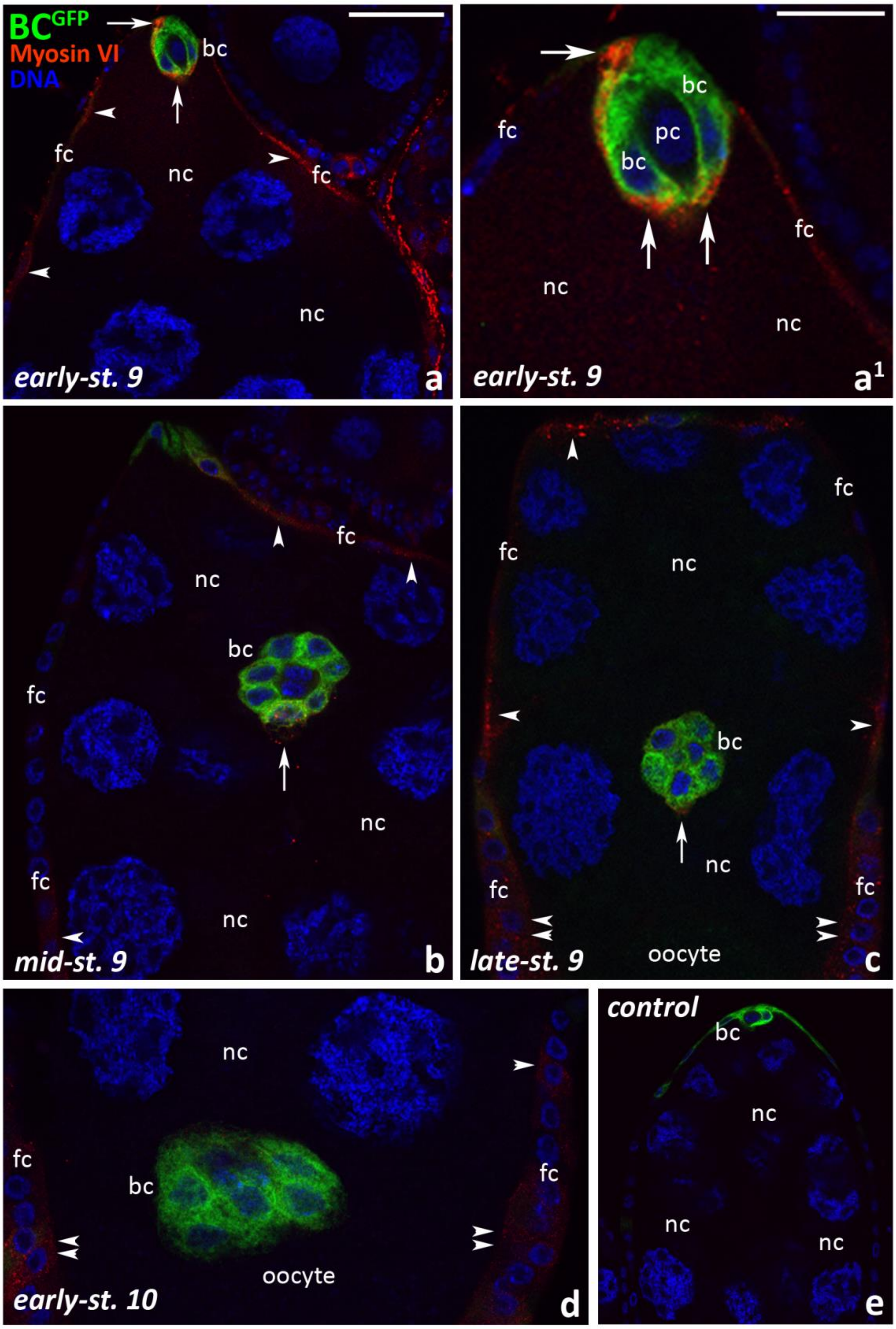

FIGURE S2. Immunocytochemical localization of myosin VI in the egg chambers of BC^{GFP} *Drosophila* females during border cell migration: the early-stage 9 (**a**, **a^1**), mid-stage 9 (**b**), late-stage 9 (**c**), and early-stage 10 (**d**); **a^1** is a bigger magnification of **a**. A negative control of immunocytochemical labeling shows a complete lack of unspecific red fluorescence signals (**e**). Border cells are stained in green, myosin VI is stained in red, and cell nuclei are stained in blue. Arrows show localization of myosin VI in the border cell cluster, arrow heads show presence of this protein in the follicular epithelium. bc, border cells; fc, follicular cells; nc, nurse cells; polar cells. Bar 25 μm (**a**, **b**-**e**), 10 μm (**a^1**).
