# Supplementary material for: Myosin VI is expressed in developing ovarian follicles in Drosophila but is not essential for effective oogenesis
Source: Front Cell Dev Biol. 2025 Jun 2;13:1535117. doi: 10.3389/fcell.2025.1535117 (PMC12171261; doi:10.3389/fcell.2025.1535117)
Supplement: Supplementary file 2 [file Image3.pdf]

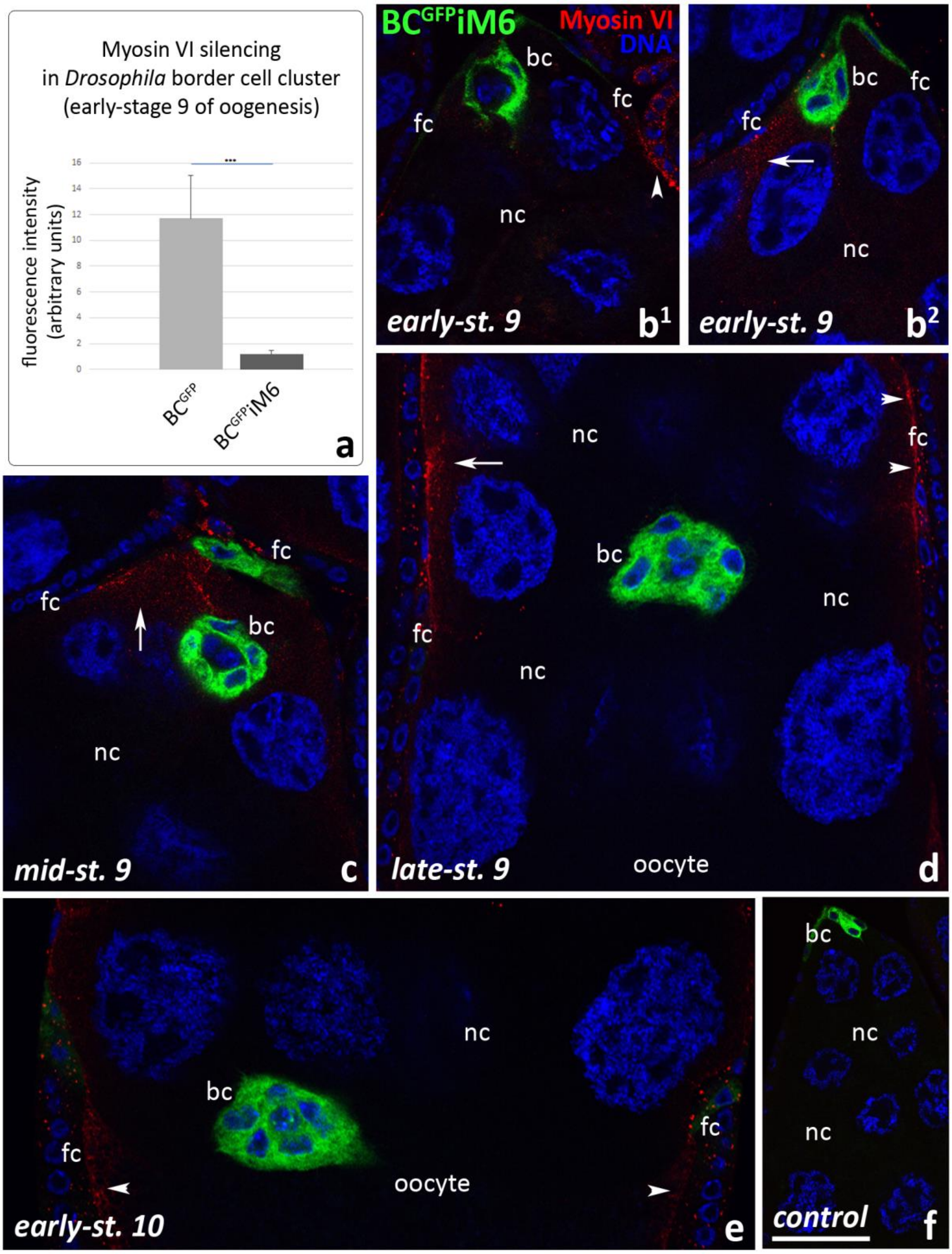

FIGURE S3. Efficiency of myosin VI gene silencing in border cells and immunocytochemical localization of myosin VI in the egg chambers of BC^{GFP}iM6 *Drosophila* females during border cell migration: the early-stage 9 (**b**, **b^{1}**), mid-stage 9 (**c**), late-stage 9 (**d**), and early-stage 10 (**d**). Quantitative analysis show that the level of myosin VI was approximately 90% lower in border cells in BC^{GFP}iM6 egg chambers compared to control chambers (**e**). Graphs (**a**) show the relative myosin VI levels in border cells (mean of 20 replicates and standard deviation). Arbitrary units on the y-axis show myosin VI-associated fluorescence intensity (pixels). Statistical analysis was carried out using the Mann–Whitney test (*** $p \leq 0.001$). A negative control of immunocytochemical labeling shows a complete lack of unspecific red fluorescence signals (**f**). Border cells are stained in green, myosin VI is stained in red, and cell nuclei are stained in blue. Arrows show the labeling in the nurse cells and arrow heads show the labeling in the follicular epithelium. bc, border cells; fc, follicular cells; nc, nurse cells. Bar 25 μm.
