# Supplementary material for: Myosin VI is expressed in developing ovarian follicles in Drosophila but is not essential for effective oogenesis
Source: Front Cell Dev Biol. 2025 Jun 2;13:1535117. doi: 10.3389/fcell.2025.1535117 (PMC12171261; doi:10.3389/fcell.2025.1535117)
Supplement: Supplementary file 3 [file Image1.pdf]

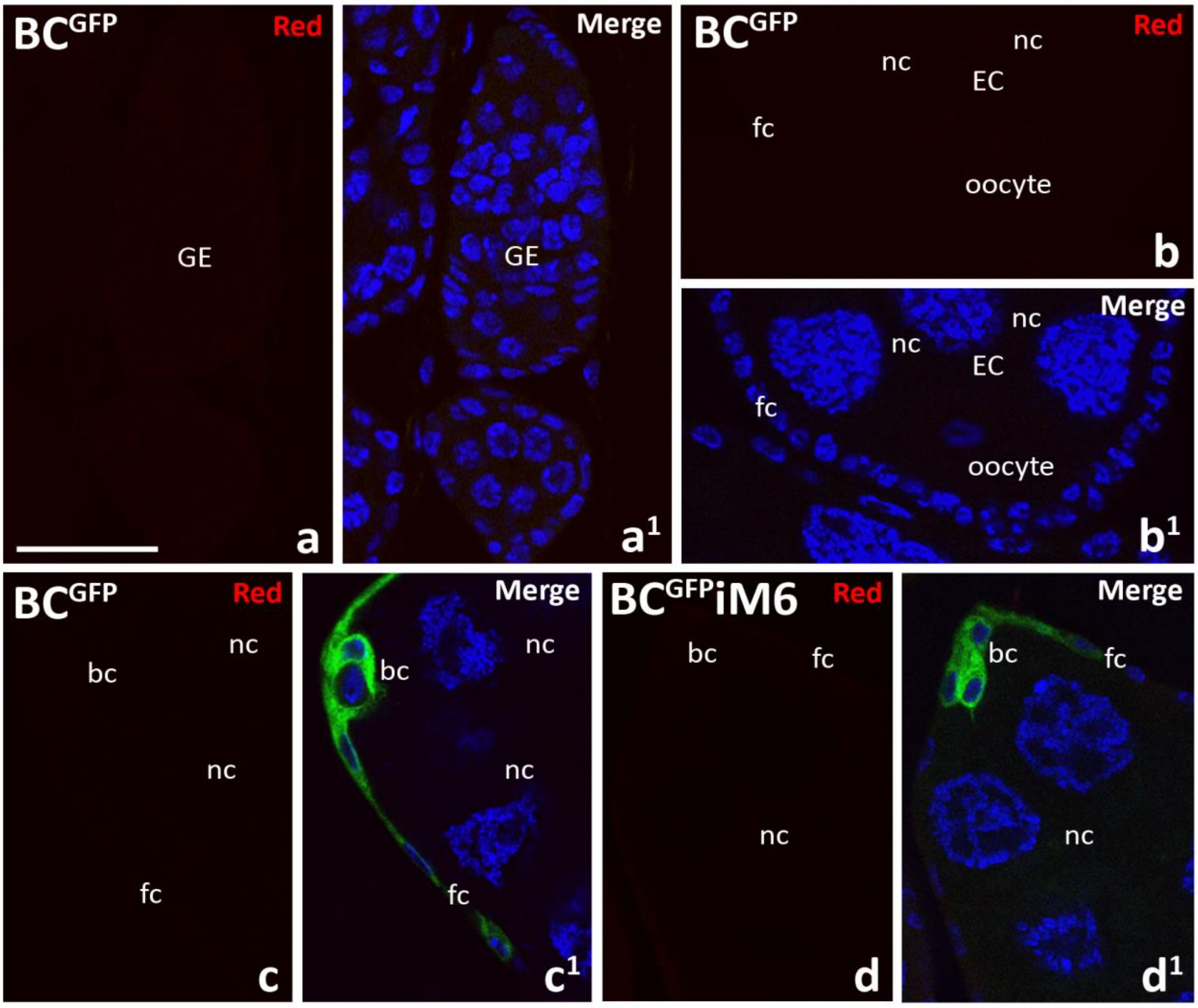

FIGURE S1. Negative control of immunocytochemical labeling in early ovarian follicles of BC^{GFP} and BC^{GFP}iM6 *Drosophila* females. A negative control omitting the primary antibody against myosin VI shows a complete lack of unspecific signals (red fluorescence) in germarium (**a**, **a^1**) and developing oocyte (**b**, **b^1**) of the BC^{GFP} females as well as in border cells at stage 8 of oogenesis of the BC^{GFP} (**c**, **c^1**) and BC^{GFP}iM6 (**d**, **d^1**) females. bc, border cells; fc, follicular cells; nc, nurse cells. Bars 25 µm.
